# Supplementary material for: Conformational dynamics and multimodal interaction of Paxillin with the focal adhesion targeting domain
Source: Sci Adv. 2025 Jun 18;11(25):eadt9936. doi: 10.1126/sciadv.adt9936 (PMC12175908; doi:10.1126/sciadv.adt9936)
Supplement: Supplementary file 1 — Figs. S1 to S14 Table S1 [file sciadv.adt9936_sm.pdf]

Science Advances
AAAS

Supplementary Materials for

Conformational dynamics and multimodal interaction of Paxillin with the focal adhesion targeting domain

Supriyo Bhattacharya *et al.*

Corresponding author: Supriyo Bhattacharya, sbhattach@coh.org; Ravi Salgia, rsalgia@coh.org; John Orban, jorban@umd.edu

Sci. Adv. **11**, eadt9936 (2025)
DOI: 10.1126/sciadv.adt9936

This PDF file includes:

Figs. S1 to S14
Table S1

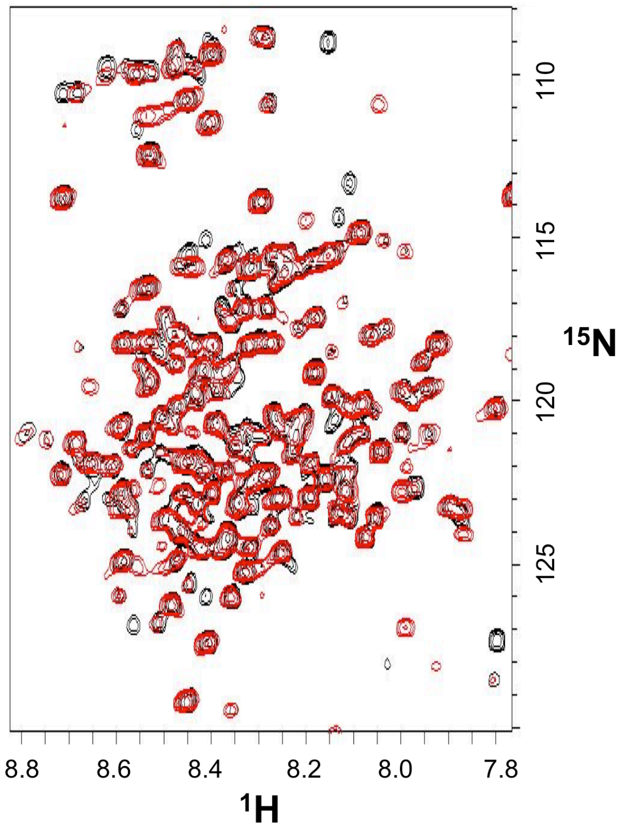

Figure S1: Comparison of full-length PXN and N-domain PXN NMR spectra. Overlaid two dimensional ^{1}H-^{15}N HSQC spectra of the 311-residue N-domain of PXN (black) and 557-residue, full-length PXN (red). Peaks due to the ordered LIM domains in full-length PXN are broadened and not readily apparent, presumably due to their slower tumbling relative to the more flexible N-domain.

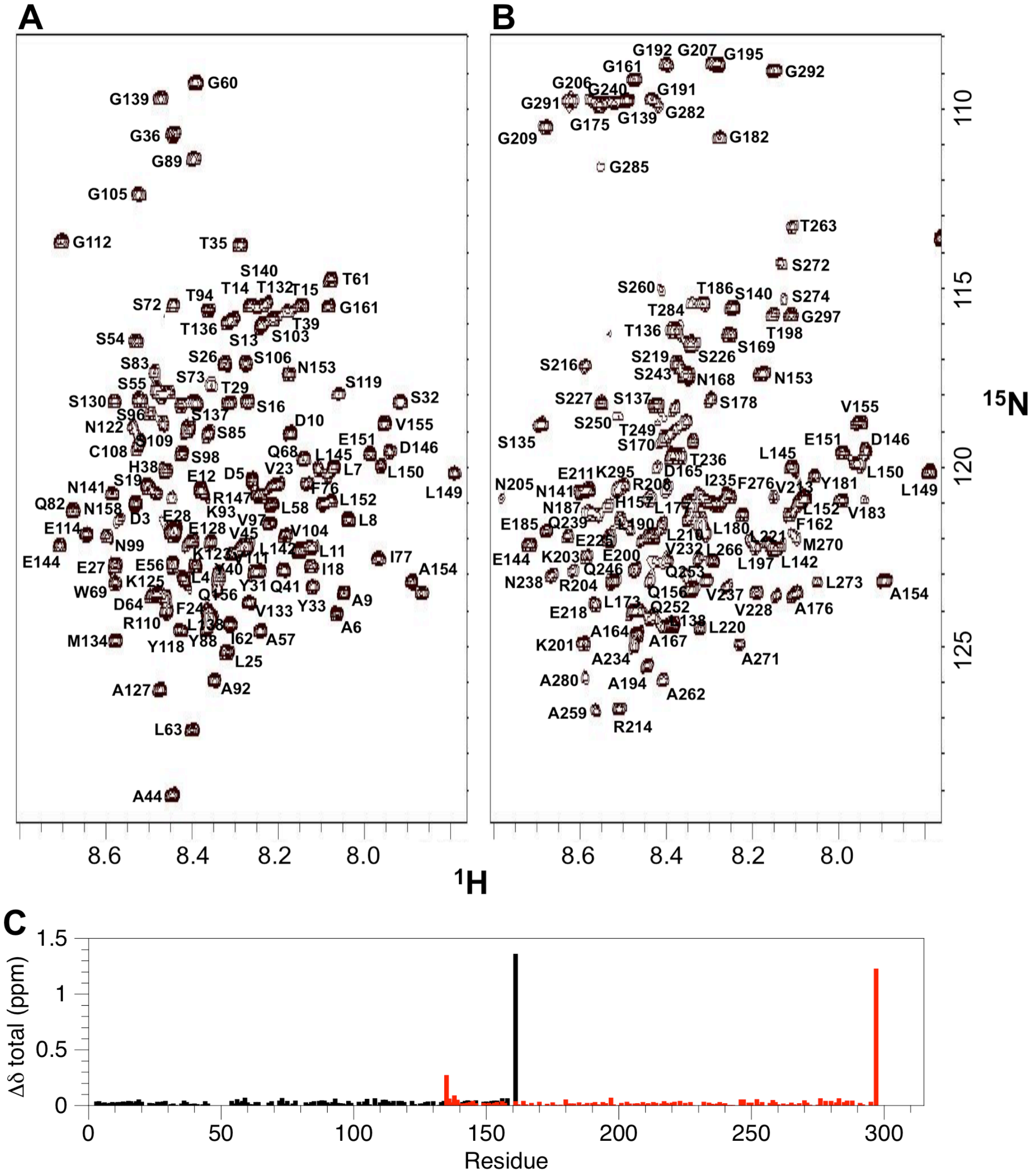

Figure S2: Backbone amide assignment of PXN fragments. Two dimensional ^{1}H-^{15}N HSQC spectra for (**A**) PXN LD1-2 and (**B**) PXN LD2-4 with backbone amide chemical shift assignments. (**C**) Backbone amide chemical shift perturbations between the PXN N-domain (LD1-5) and the corresponding residues in LD1-2 (black) and LD2-4 (red). The chemical shift perturbations were determined using $\Delta\delta_{total} = [(W_H\Delta\delta_H)^2 + (W_N\Delta\delta_N)^2]^{1/2}$, where $W_H = 1$ and $W_N = 0.2$

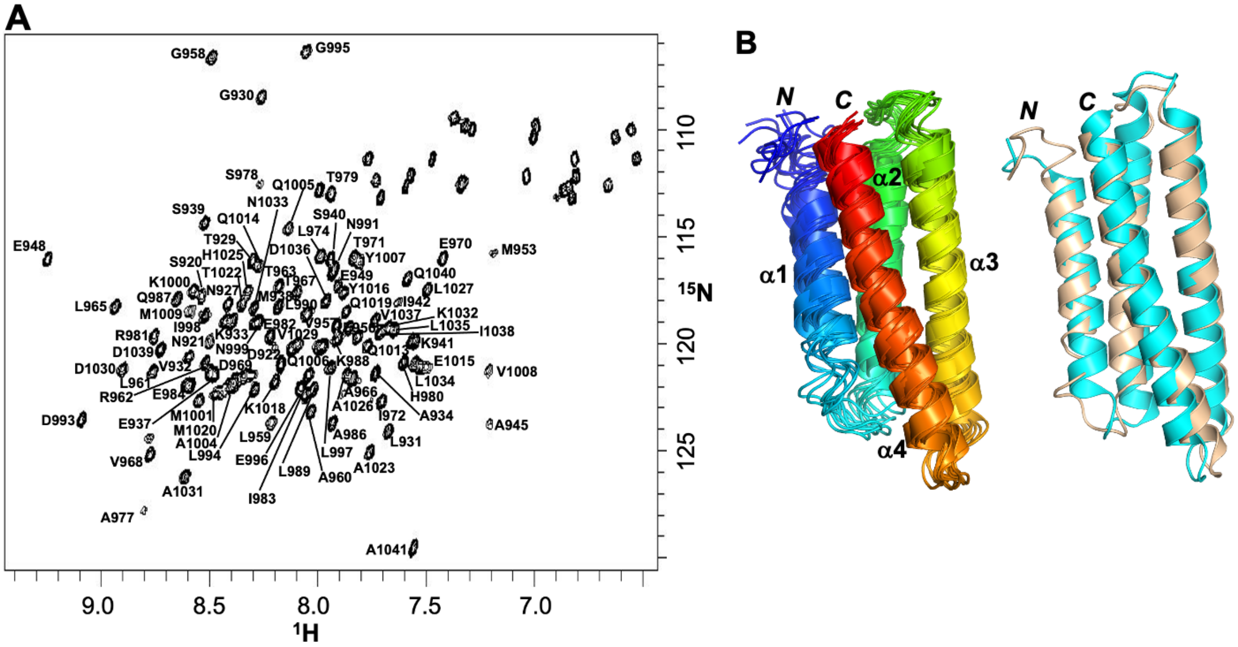

Figure S3: **NMR structural analysis of human FAT.** Structural analysis of human FAT. (**A**) Two dimensional ^{1}H-^{15}N HSQC spectrum of human FAT with backbone amide peak assignments. (**B**) CSRosetta structure showing the ensemble for the 10 lowest energy conformations (left), deposited in PDBDev (Accession code 00000391). Superposition with the X-ray structure (PDB 1OW8, cyan) gives a backbone RMSD of 1.5 Å (right). See Table S1 for structure statistics.

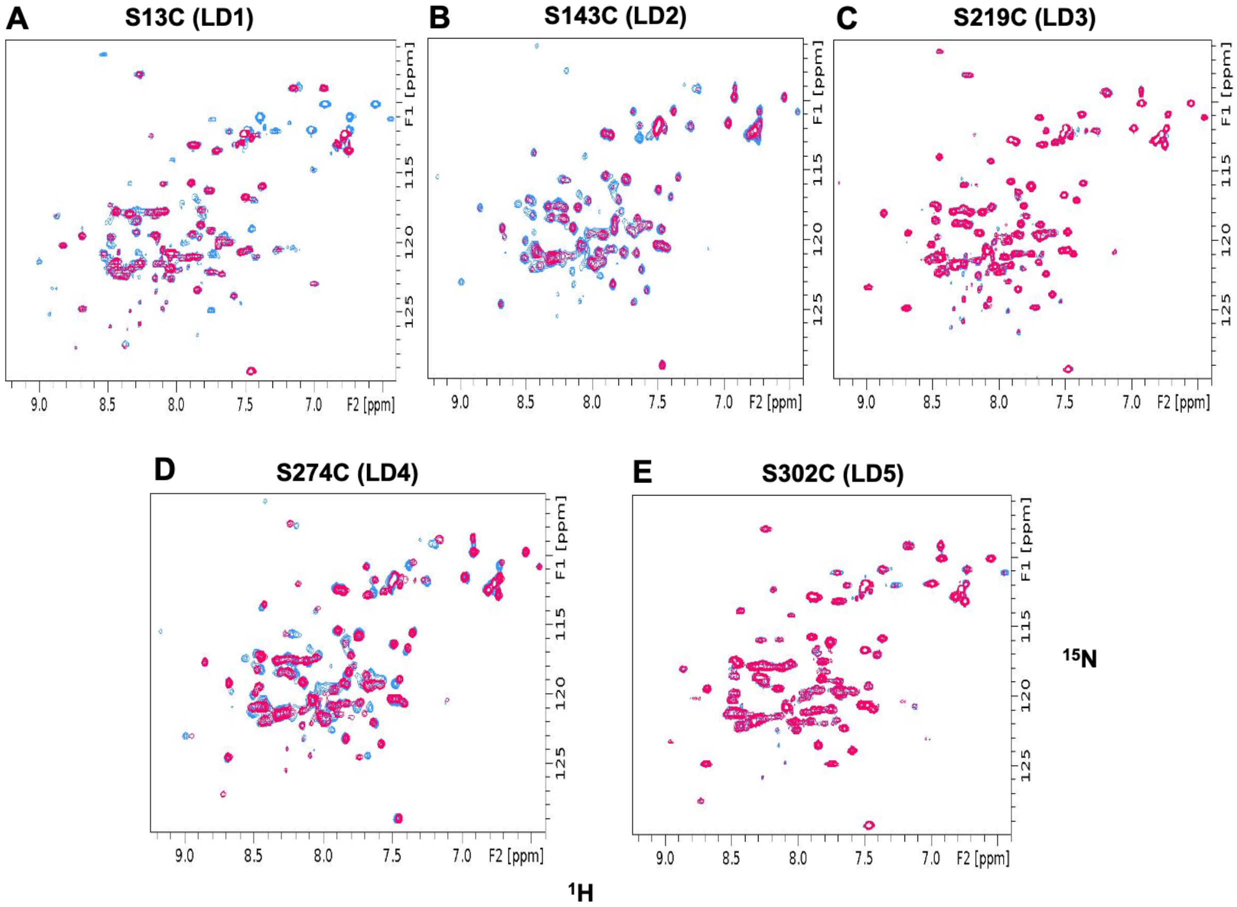

Figure S4: Intermolecular PRE data for determining how PXN binds FAT. Two dimensional ^{1}H-^{15}N HSQC spectra of the FAT domain used for mapping binding epitopes of LD motifs onto the FAT surface, as described in Figure 4. Overlaid spectra for reduced (blue) and oxidized (red) states are shown for each PXN MTSL-spin label position as indicated.

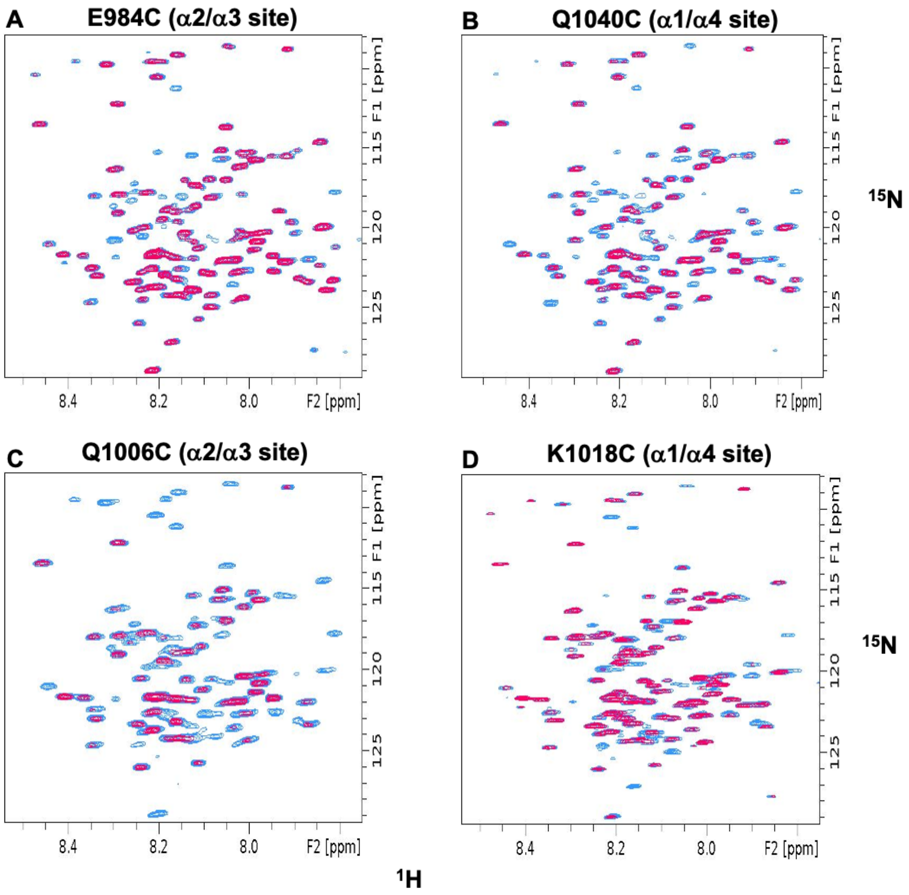

Figure S5: Intermolecular PRE data probing flexibility of the FAT-bound PXN chain. Two dimensional ^{1}H-^{15}N HSQC spectra of the PXN N-domain used to probe its conformational dynamics around the α2/α3 and α1/α4 sites of the FAT domain, as described in Figure 5. Overlaid spectra for reduced (blue) and oxidized (red) states are shown for each FAT MTSL-spin label position as indicated.

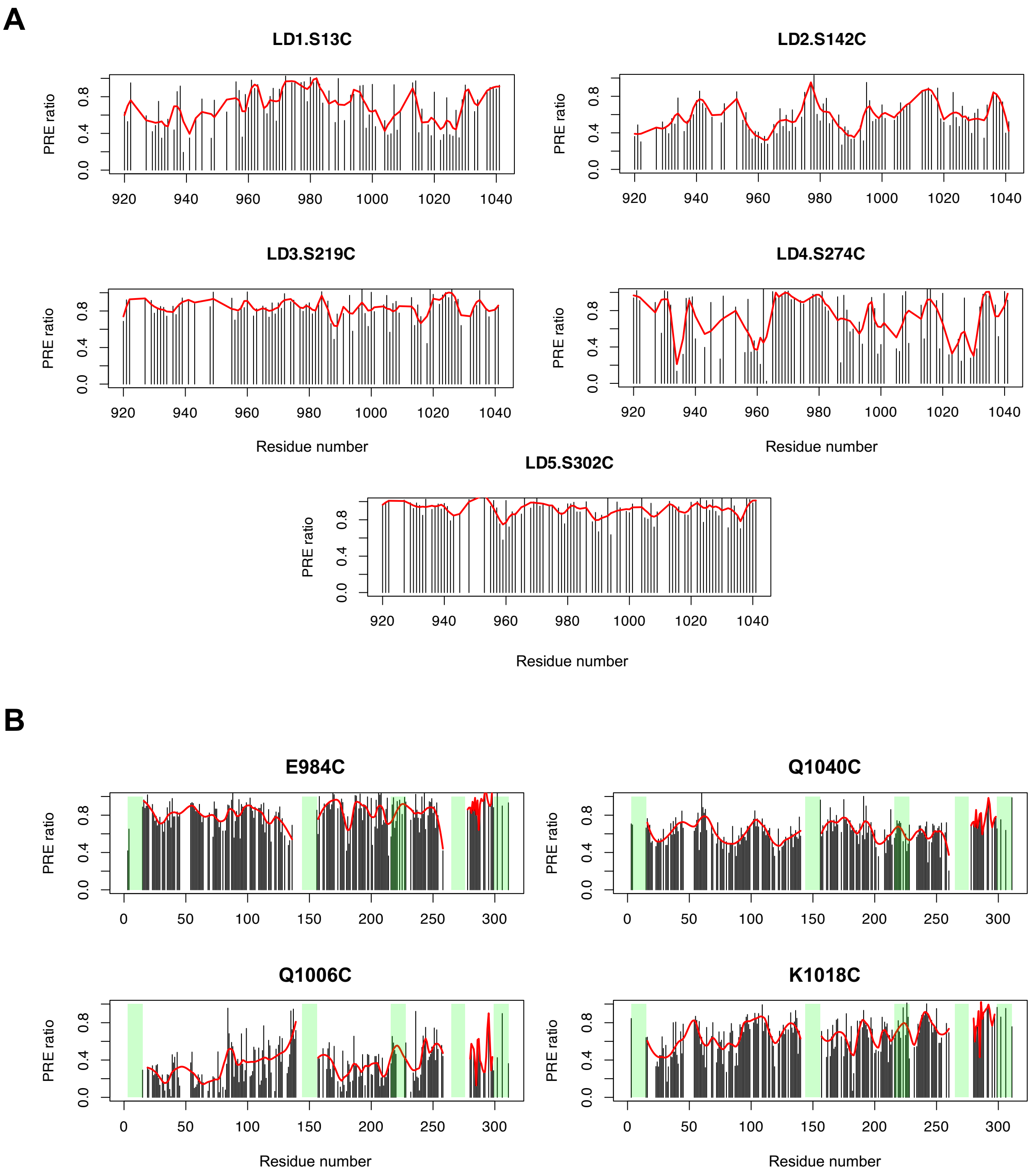

Figure S6: Comparison of smoothed and unsmoothed intermolecular PRE profiles. Smoothing of the experimental PRE profiles using the LOESS algorithm for MTSL probes along (**A**) PXN sequence, (**B**) FAT sequence. Black lines represent the original data and the red curves the smoothed profiles. For each plot in panel B, the positions of the LD motifs are highlighted in green.

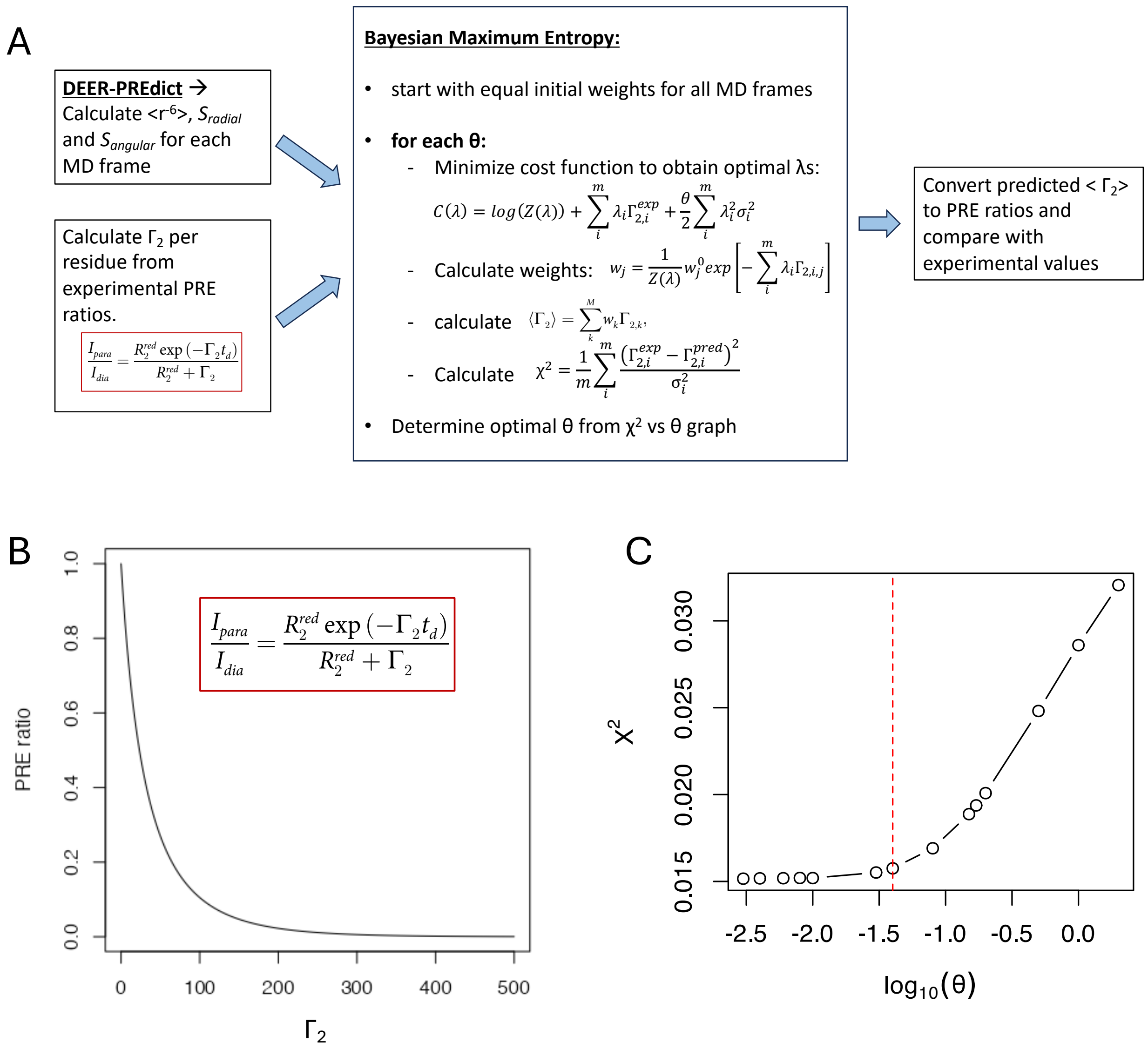

Figure S7: Computational pipeline for deriving trajectory frame weights using experimental PRE ratios. (**A**) Schematic describing the iterative BME procedure for deriving trajectory weights in conjunction with experimental PRE ratios. (**B**) Relationship between Γ_2 and PRE intensity ratio, as reflected by the equation in the red box. (**C**) Variance scaled mean square error χ^2 as function of θ. The optimal θ is marked by the vertical red dashed line.

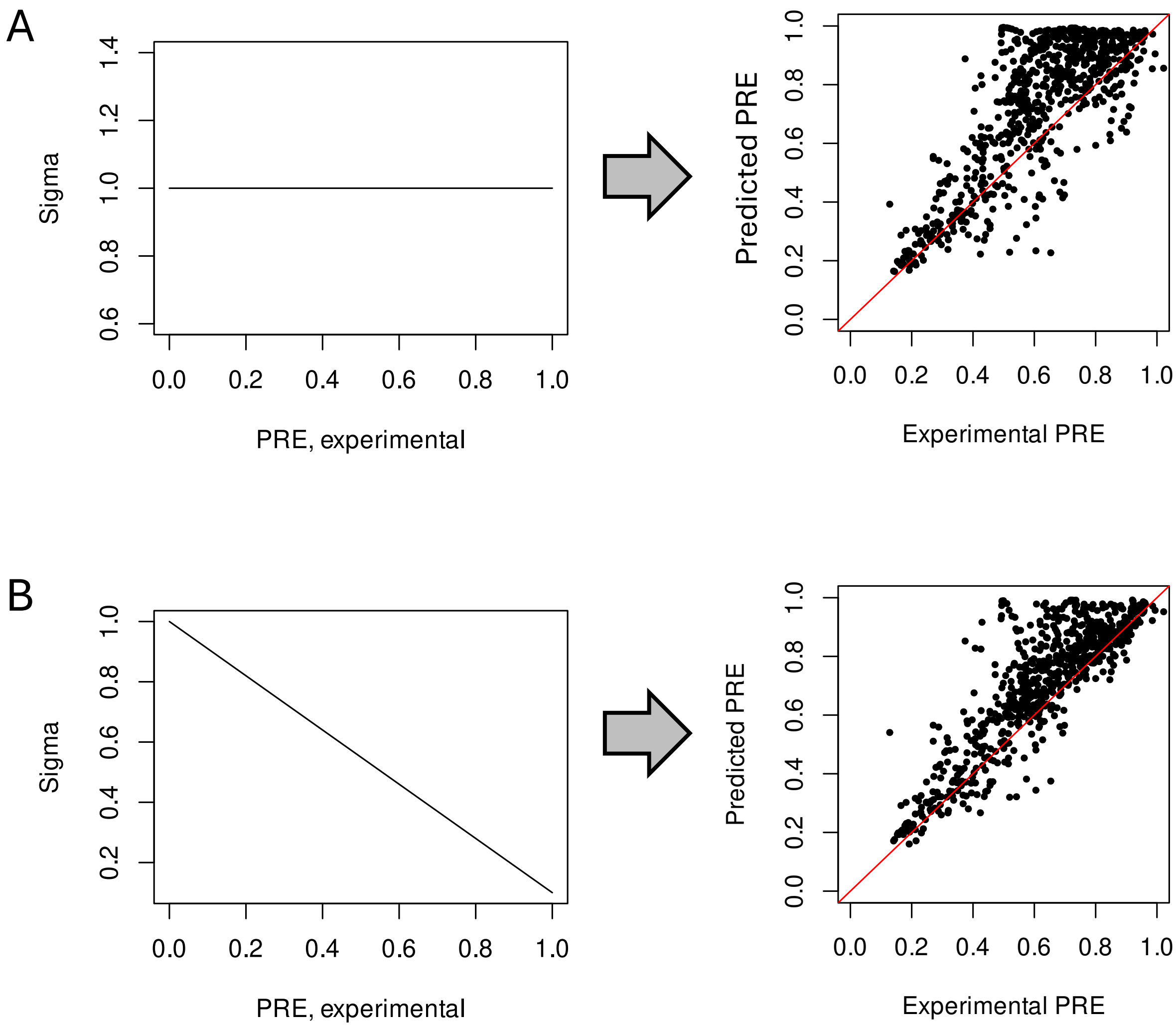

Figure S8: **Applying variable sigma in deriving trajectory frame weights using the BME method.** Comparison of PRE correlations obtained using constant (**A**) versus variable sigma (**B**) in the BME equation (see Methods).

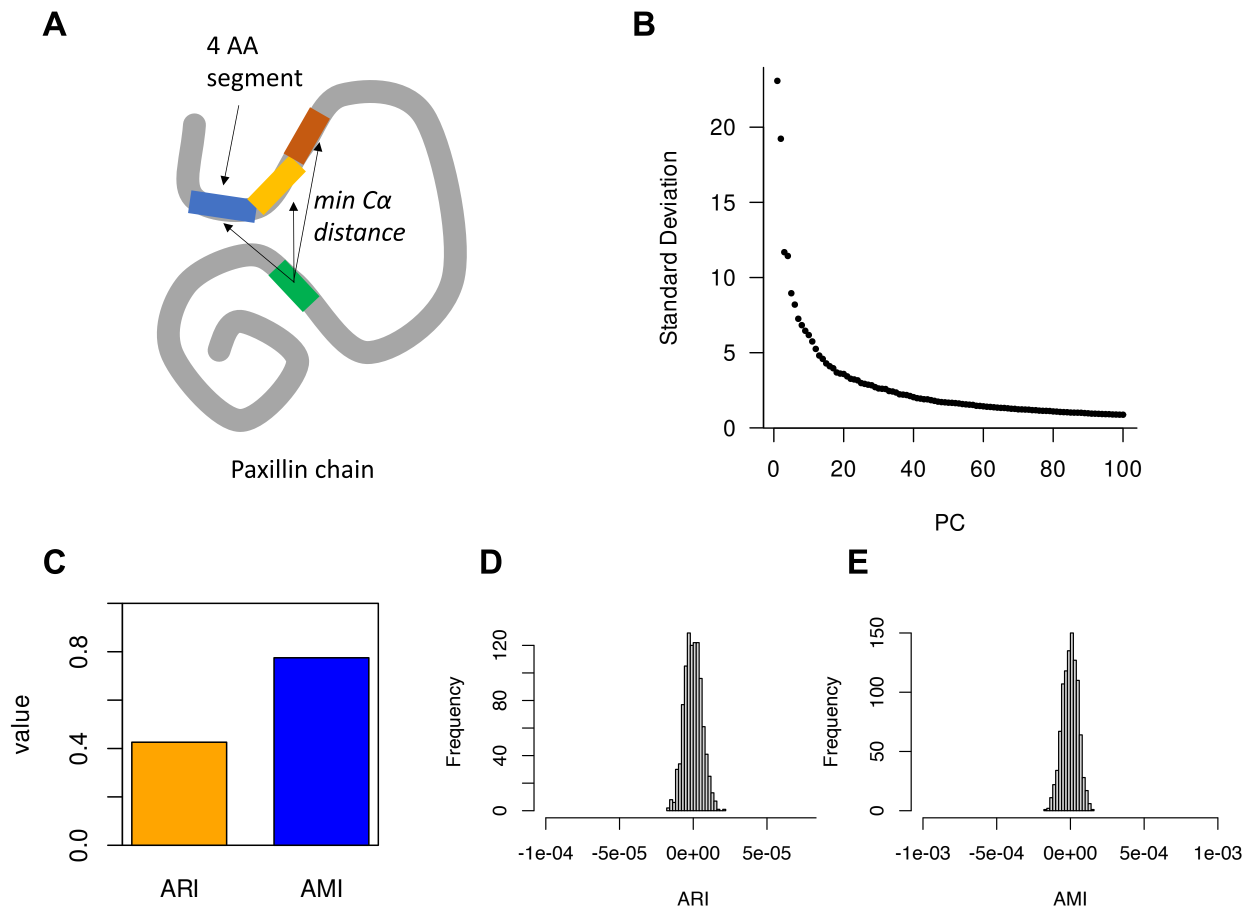

Figure S9: Structure-based similarity metric for clustering PXN MD conformations and UMAP projection. (**A**) Schematic describing the amino acid segments along the PXN chain used in calculating inter-segment distances for deriving the UMAP. (**B**) Percentage of variation explained by the top 100 principal components (PCs) as calculated from the inter-segment distances among the trajectory frames. (**C**) Adjusted Rand Index (ARI) and Adjusted Mutual Information (AMI) between the conformation clusters obtained using the top PCs versus the two UMAP coordinates. (**D-E**) Null bootstrapped ARI and AMI distributions obtained by randomly scrambling the UMAP-based cluster labels 10,000 times and comparing with the PC-based cluster labels.

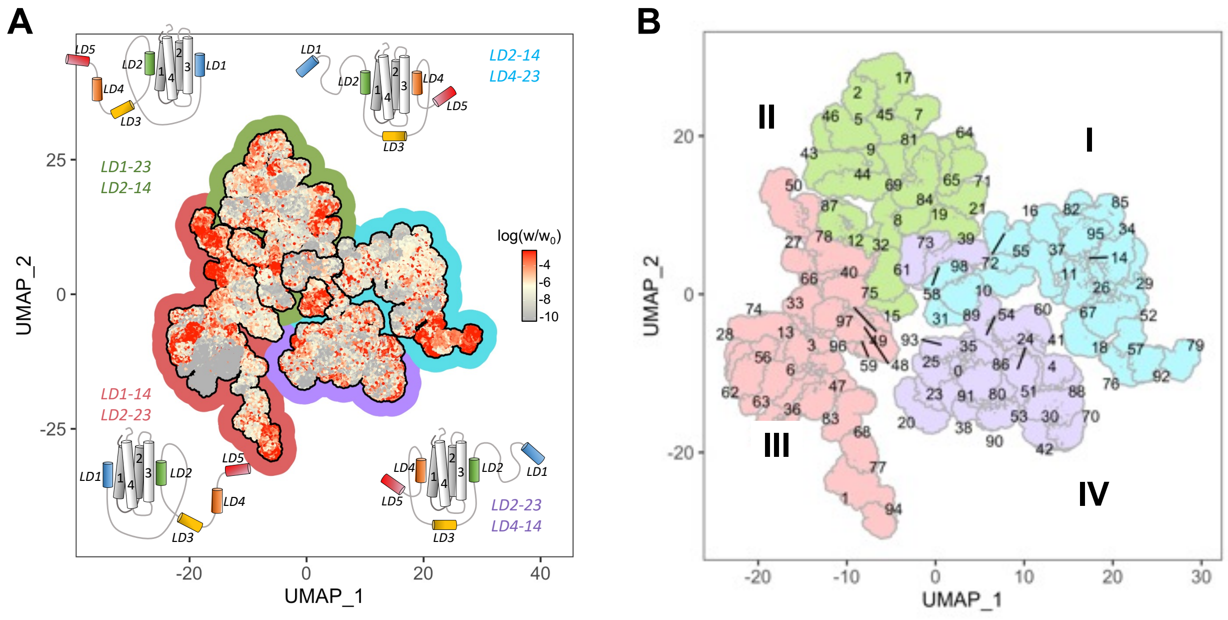

Figure S10: UMAP representation of PXN conformational ensemble. (**A**) MD-generated PXN/FAT ensemble projected in UMAP space, where the UMAP coordinates are derived using PXN inter-chain contacts in each MD conformation (see Methods for details). The conformations are color-coded according to their weights obtained using the BME approach. Red regions in the UMAP contribute more towards the PRE agreement compared to gray regions. Colored halos around each UMAP region are indicative of the PXN/FAT orientation used in the MD simulations. (**B**) MD-derived PXN conformations shown in a UMAP plot. Clusters are labeled using cluster indices, gray lines demarcate cluster boundaries, and conformations are color-coded according to the PXN orientation (I-IV).

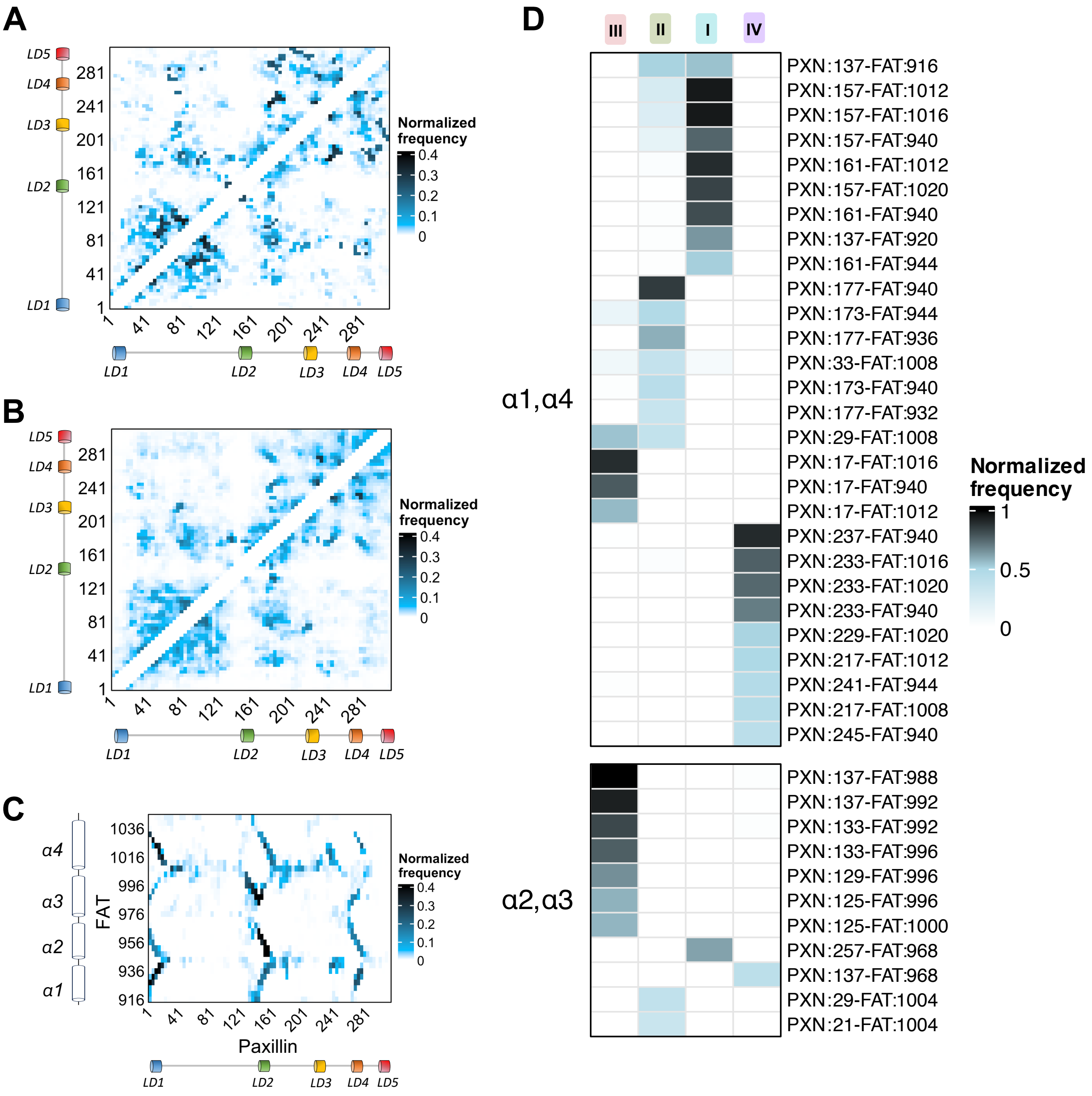

Figure S11: PXN-FAT interaction frequency map from reweighted MD ensemble. (**A**) Contact map of the FAT-bound PXN N-domain conformational ensemble obtained from MD using the BME reweighted ensembles. The PXN N-domain was divided into consecutive 4 amino acid-long segments and the minimum Cα distances between segment pairs were calculated for each MD frame. Two segments were defined to be in contact if their inter-segment distance was less than 8Å (for details, see Methods and Fig. S9A). Cells in the contact map are colored according to the contact frequencies of

segment pairs. Short range contacts between consecutive segments were omitted. (**B**) Contact map derived from the original unweighted MD ensemble. (**C**) PXN/FAT contact map using the BME reweighted PXN ensemble. The contact definition is the same as for panels A-B. (**D**) Heatmap depicting the top PXN/FAT linker region contacts per MD-derived state, shown separately for the α1/α4 and α2/α3 faces of the FAT domain.

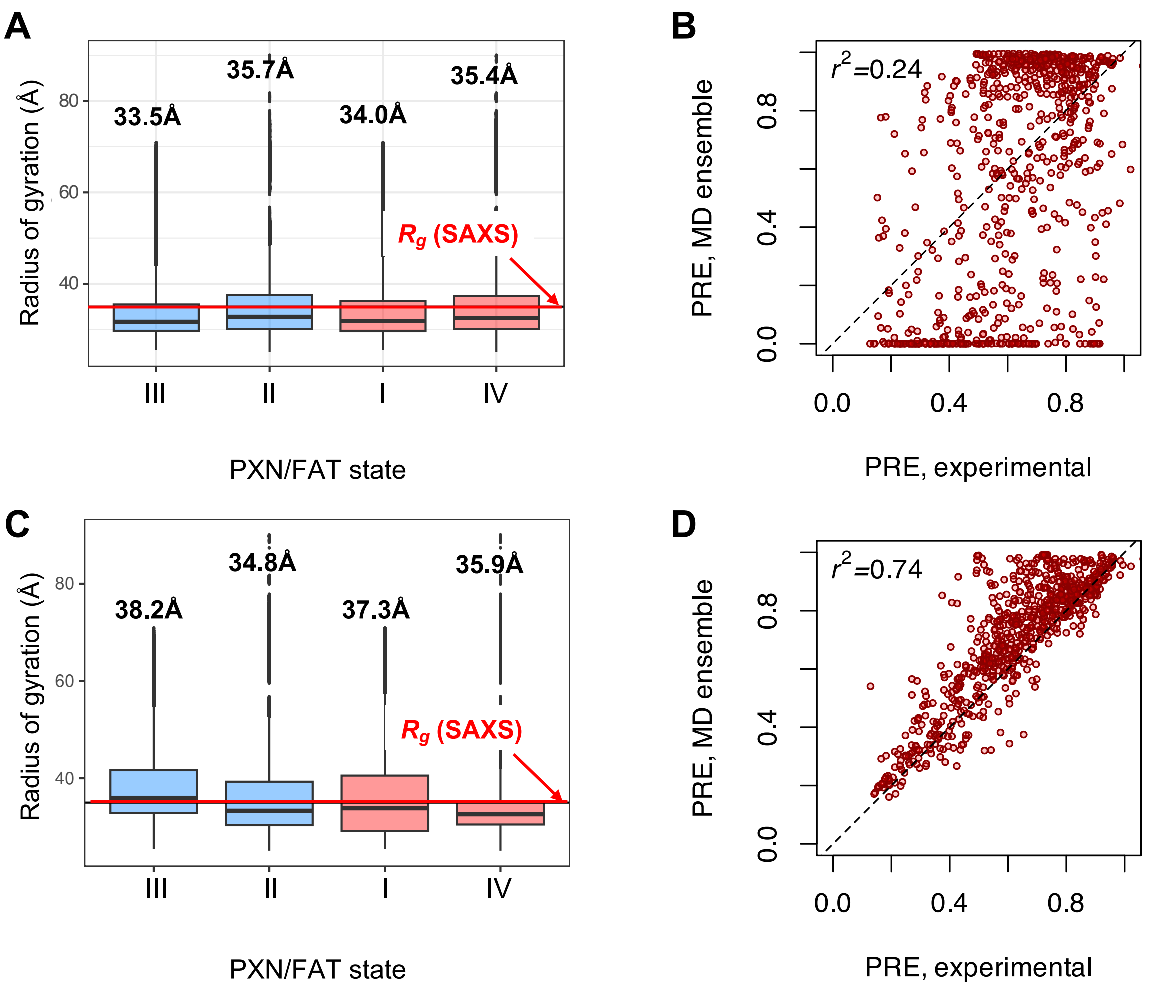

Figure S12: Radii of gyration and PRE correlations from PXN MD ensembles. Radii of gyration and PRE agreement comparison between the original unweighted **(A,B)** and BME reweighted **(C,D)** ensembles, for each of the four PXN/FAT states. **(A, C)** Box and whisker plots showing the radii of gyration (R_g) calculated from the original and BME reweighted ensembles respectively. Boxes represent the interquartile ranges, with the outlier MD conformations plotted along the vertical lines above and below each box. The horizontal red line is the experimental R_g (35Å) obtained from SAXS. **(B,D)** PRE intensity ratios from all four MTSL probes are compared between their experimental and MD derived counterparts, for the original **(B)** and reweighted **(D)** ensembles. Pearson's correlation coefficients (r^2) are given in the plots.

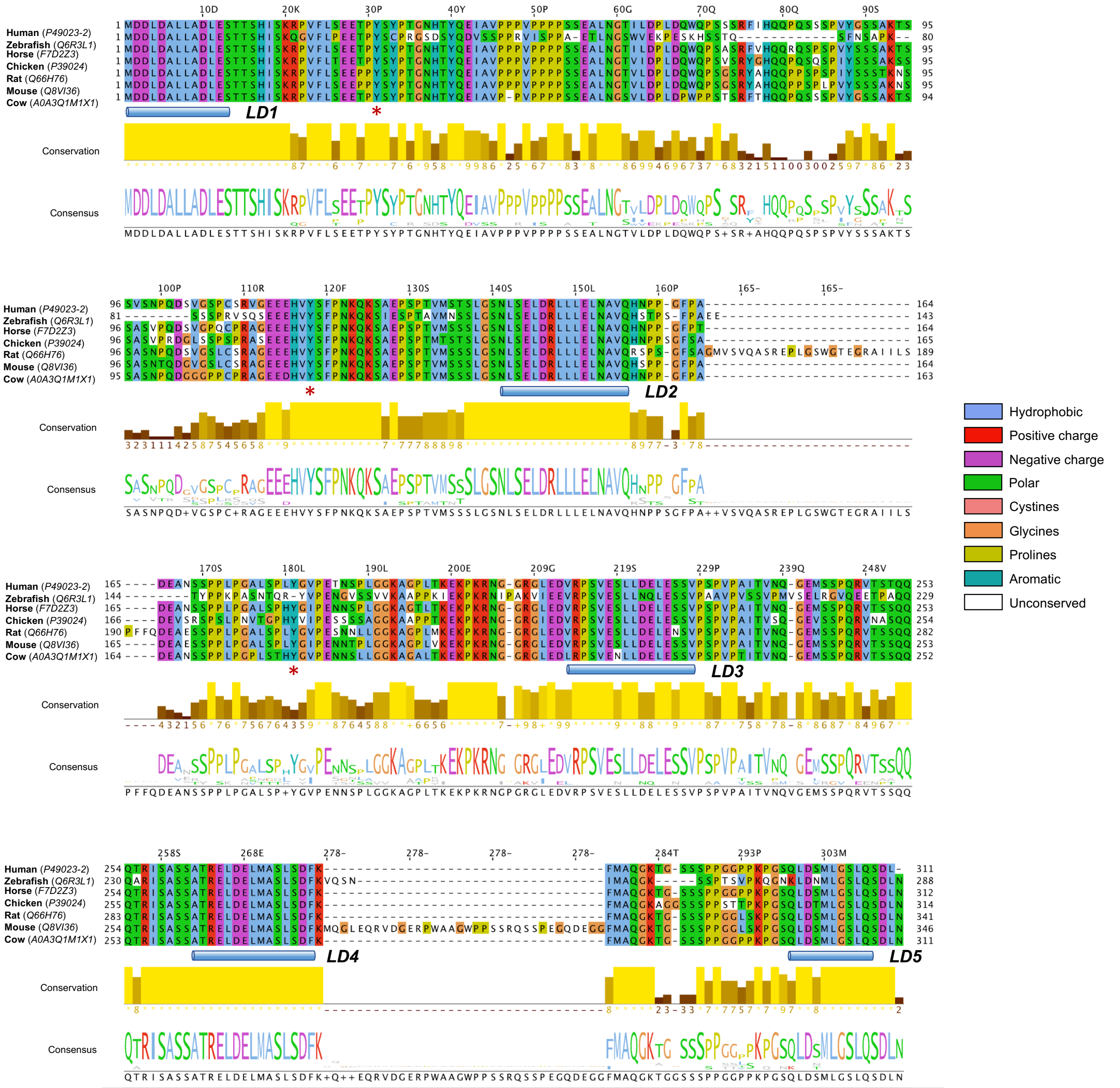

Figure S13: **Sequence conservation of PXN from multiple species.** Multiple sequence alignment of PXN from different species showing the conservation status of the intrinsically disordered regions. Sequences are labeled by the name of the species in bold, followed by the Uniprot ID in brackets. Residue positions are colored according to amino acid type as indicated in the figure. Conservation scores are represented by the yellow bar plot, where lighter shades of yellow indicate more conserved regions. The consensus sequence is highlighted using amino acid logo. The positions of the LD motifs are marked by the blue cylinders. Locations of the three tyrosine phospho-sites are marked by red star symbols.

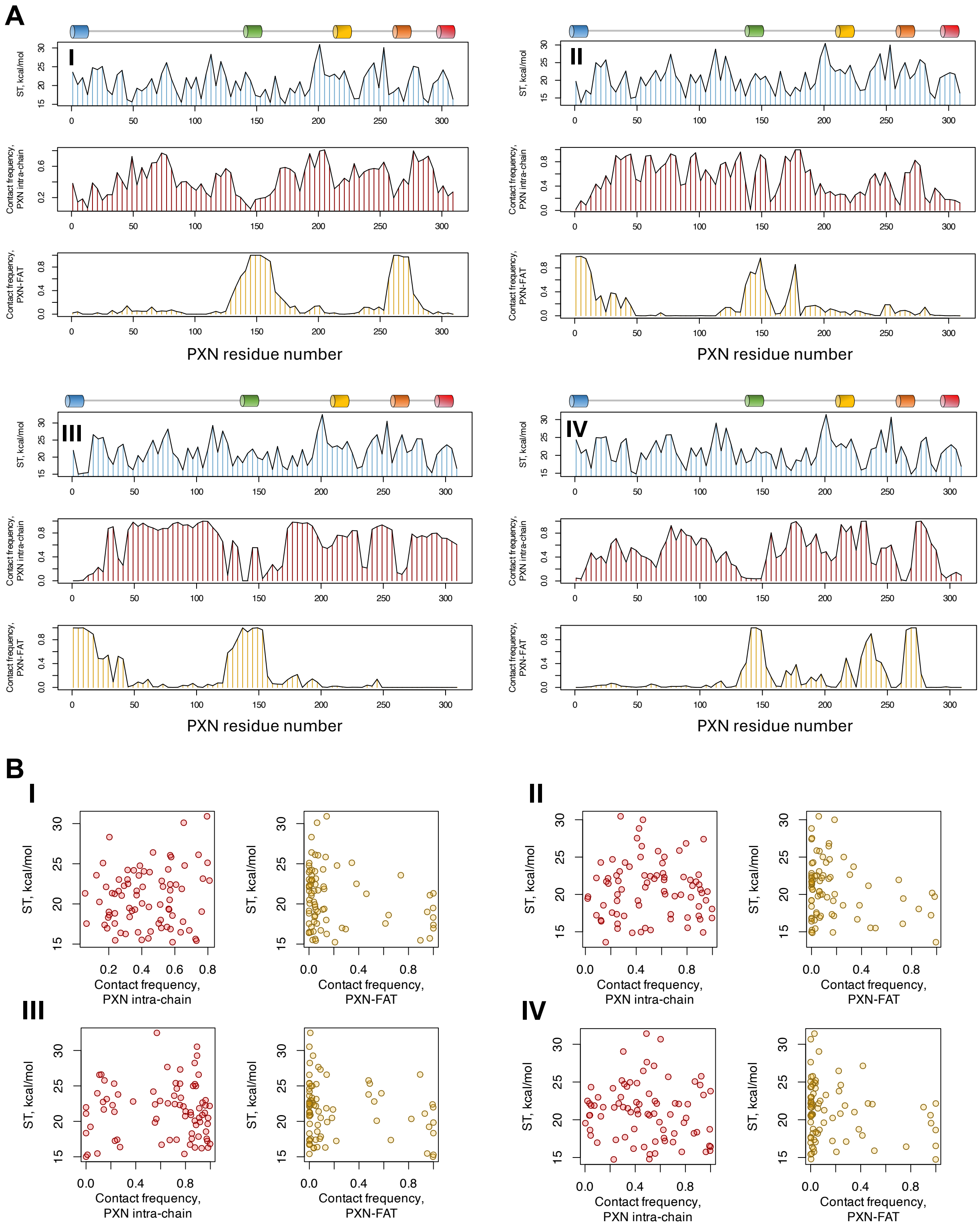
A
I
II
III
IV
ST, kcal/mol
Contact frequency, PXN intra-chain
Contact frequency, PXN-FAT
PXN residue number
B
Contact frequency, PXN intra-chain
Contact frequency, PXN-FAT

Figure S14: **Conformational entropy of the disordered linker regions in PXN** (**A**) Residue-wise entropy (blue), intra-chain (red) and PXN-FAT (yellow) contact frequencies are plotted as function of PXN sequence for all four configurations. Entropy unit is the same as described in Fig. 10. Locations of the LD helices are shown above each plot using the same color convention that is throughout the manuscript. (**B**) Intra-chain (red dots) and PXN-FAT (yellow dots) contact frequencies for PXN residues plotted against their entropy values separately for the four configurations.

Table S1: Statistics for 10 best FAT structures

A. Experimental chemical shift inputs	
$^{13}C\alpha$	113
$^{13}C\beta$	110
^{13}CO	109
^{15}N	95
$^{1}H_N$	95
$^{1}H\alpha$	84
B. RMSDs to the mean structure (Å)	
Over all residues [a]	
Backbone atoms	2.17 ± 0.52
Heavy atoms	2.72 ± 0.59
Secondary structures [b]	
Backbone	1.86 ± 0.50
Heavy atoms	2.41 ± 0.56
C. Measures of structure quality	
Ramachandran distribution (%) [c]	
Most favored	96.2 ± 2.1
Additionally allowed	3.8 ± 2.1
Generously allowed	0.0 ± 0.0
Disallowed	0.0 ± 0.0
D. PDB/BMRB codes	
PDBDEV	00000391
BMRB	51556

[a] Residues 918-1041.
[b] The secondary elements used were as follows: residues 920-940 (α1), 947-975 (α2), 977-1006(α3), 1012-1040 (α4).
[c] Ramachandran distributions were measured with Procheck.
